# Recurrence of genitals warts in pre-HPV vaccine era after laser treatment

**DOI:** 10.1007/s00404-019-05242-5

**Published:** 2019-07-08

**Authors:** Andreas Widschwendter, Bettina Böttcher, David Riedl, Serab Coban, Irene Mutz-Dehbalaie, Raffaella Matteucci Gothe, Alexandra Ciresa-König, Christian Marth, Siegfried Fessler

**Affiliations:** 1 Department of Obstetrics and Gynecology, Medical University of Innsbruck, Anichstrasse 35, 6020 Innsbruck, Austria; 2 Department of Gynecological Endocrinology and Reproductive Medicine, Medical University of Innsbruck, Anichstrasse 35, 6020 Innsbruck, Austria; 3 University Clinic of Medical Psychology, Medical University of Innsbruck, Anichstrasse 35, 6020 Innsbruck, Austria; 4 Department of Public Health, Medical Informatics and Technology, Health Services Research and Health Technology Assessment, UMIT University for Health Sciences, Eduard-Wallnöfer-Zentrum 1, 6060 Hall i.T, Austria

**Keywords:** Human papilloma virus, HPV, Recurrence, Genital warts, Carbon dioxide laser, Multifocal lesions

## Abstract

**Purpose:**

Human papillomavirus (HPV) can cause condylomata acuminata, also known as genital warts. Our aim was to evaluate the long-term recurrence of genital warts after primary carbon dioxide laser treatment before the introduction of the vaccination against HPV.

**Methods:**

Recurrence rate and localization of genital warts were analysed in a retrospective study in 1798 women presenting with a new diagnosis of genital warts from 1992 to 2009 at a University hospital and had received laser treatment. Additionally, data on topography, pregnancy status, and cervical smear were available for women treated from 2003 to 2009 (*n* = 825, data subset 1) and systematic follow-up data for women treated in 2006 and 2007 (*n* = 242, data subset 2).

**Results:**

Median time from laser treatment to first recurrence was 14.6 weeks (data subset 2). The site most affected was the vulva (90.7%) followed by the perineum/perianal region (59.3%) and the vagina (47.3%). Abnormal Pap smear was observed in 22.6%. Systematic follow-up with a median follow-up time of 3.1 years revealed at least one recurrence in 68 (28.1%) of 242 women. Women with multifocal genital warts had a 2.9 times increased risk for recurrence compared to women with unifocal lesions (*p* = 0.01).

**Conclusions:**

Nearly 30% of women presenting with genital warts experienced at least one recurrence after treatment with carbon dioxide laser. Multifocal lesions are the strongest indicator of recurrence. These data provide an important insight to recurrence rates of genital warts before HPV vaccination and underline the significance of a long-term follow-up and HPV vaccination.

## Introduction

Human papillomavirus (HPV) infection is responsible for the development of condylomata acuminata, also known as genital warts. The most common HPV types are 6 and 11 and are found in more than 90% of genital warts [1–3]. A placebo- controlled vaccination study revealed that the time from initial HPV infection to the development of clinically visible genital warts is two to 50 months with a median of 25 months [2].

Genital warts are a significant public health problem. The reported incidence in men and women varies between 62 and 229/100,000/year [4, 5]. Genital warts are most often diagnosed in the age group 16–24 years, accounting for up to 50% of new cases of genital warts [6–8]. A diagnosis of genital warts is associated with a significant detriment to health-related quality of life [9, 10] and can cause psychosocial stress, resulting in decreased self-esteem and feelings of shame [11].

A wide range of treatment options for genital warts is available including topical, surgical, destructive and immunomodulatory regimens [12]. Efficacy of treatment varies between 22 and 94% [13] according to treatment. Complete clearance of genital warts with topical treatments like podophyllotoxin and imiquimod 5% cream varies between 35 and 83% with recurrence rates of 6–55%, respectively [14]. Advantages of laser vaporisation of genital warts is that all lesions can be treated in a single session, it achieves good cosmetic results without scar formation in almost all cases [15], allows precise tissue ablation and can be performed on lesions at any site including vaginal and cervical genital warts. Studies investigating the efficacy of laser treatment report persistent complete clearance of genital warts in 22–93% of patients [16–20].

Data of recurrence rates in the pre-vaccination era [21] are limited but are at least 20% within the first 12 weeks after primary treatment with different modalities [13]. A retrospective Canadian study described a recurrence rate of 48.5% in a high-risk population treated with cryotherapy with a median time to the first episode of 3.97 years [21].

Vaccination against HPV contributes to a decline of the presence of genital warts, especially in women being vaccinated at young age [22, 23].

The aim of this study was to determine the burden of disease in women due to recurrence of genital warts after primary carbon dioxide laser treatment before (and shortly after) HPV vaccination recommendation in girls in Austria.

## Materials and methods

### Participants

This retrospective study includes women who presented with a new diagnosis of genital warts at the Department of Obstetrics and Gynecology, Medical University of Innsbruck, Austria, during the period January 1992 to December 2009 (18 years, *n* = 1798). Excluded were women who were immunocompromised, including HIV infection. None of the patients, even after 2006, was vaccinated against HPV. Diagnostic procedure included gynecological examination and Pap smear. Postoperative controls (four weeks after operation) and follow-up were performed by a specialised gynecologist practising outside the institution. In the event of recurrence women were usually sent back to our institution for additional laser treatment.

### Data collection

Data collection was divided in one main data set und two subsets.

The main data set (data set 1) consisted of all patients who presented with a diagnosis of genital warts between 1992 and 2009 (*n* = 1798).

The first subset (data set 2) consisted of data on topography of genital warts, pregnancy status and cervical smear being available for women treated from 2003 to 2009 (*n* = 825). Pap smears were classified according to the Second Munich Nomenclature [24]. Location of lesions was retrieved from surgical report.

The second subset (data set 3) consisted of data of a systematic follow-up data following primary treatment until December 31st 2011. These data including a thorough gynecologic examination were available for women treated in 2006 and 2007 (*n* = 242) and were retrieved from each patient’s gynaecologist and/or general practitioner. Gynecologic inspections were performed four weeks after primary treatment, and thereafter every 6–12 months.

### Statistical methods

Standard methods of descriptive statistics (median, minimum, maximum, range, frequencies) were used. Associations between categorical variables were tested with Pearson’s chi-square test. The Mann–Whitney *U* test was used to assess differences between the means of independent groups in case of non-normally distributed variables. The Kaplan–Meier method was used for univariate analysis, and the log rank test to assess the difference between recurrence curves. The Cox proportional hazards analysis was used to estimate hazard ratios and corresponding 95% confidence intervals for various prognostic variables. The multivariate analysis included patients’ age, location of genital warts (dichotomous variables for vagina, vulva, perineum/perianal region, and anus) and number of affected sites (dichotomous variable: unifocal vs multifocal) as independent variables. A *p* value < 0.05 was considered statistically significant. All statistical analyses were performed with SPSS, version 22.0 (IBM Corporation, Armonk, NY).

### Ethical approval

The study was approved by the Ethics Committee of Medical University Innsbruck (reference number 4198/2011).

## Results

**Fig. 1:**
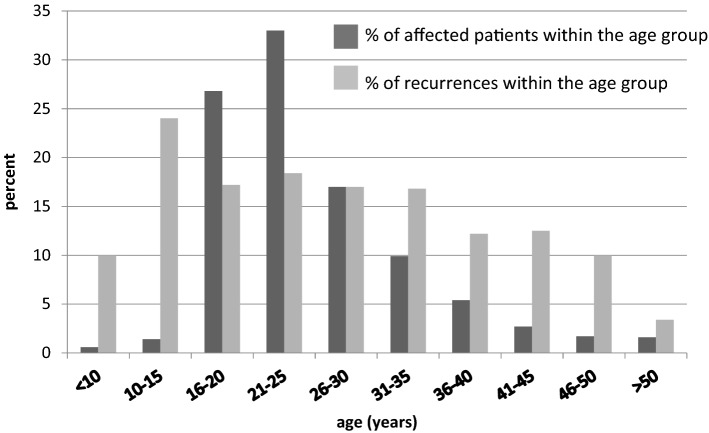
Age group and frequency of recurrence of genital warts in girls/women in percentage (*n* = 1798)

From 1992 to 2009 a total of 1798 women with genital warts were treated with carbon dioxide laser vaporisation and with electrocoagulation in the case of additional anal warts. From 1992 to 2000 an average of 83 women with genital warts were treated per year, whereas from 2001 to 2009 a 42% increase in women with genital warts (average: 117 patients per year) was observed. Median age of our patients was 24.0 (range 2.0–74.0) years. Primary diagnosis of genital warts was most frequent in women between 21 and 25 years of age, followed by the age groups 16–20 years and 26–30 years (Fig. 1).

### Percentages of recurrence

At least one recurrence was observed in 306 (17.0%) of the 1798 study patients. Two recurrences occurred in 3.2%, three recurrences in 1.1% and nine women (0.5%) had more than three recurrences (Table 1).

**Table 1:** Frequency of recurrence in all women (primary diagnosis of genital warts between 1992 and 2009), women with primary diagnosis between 2003 and 2009 and women with primary diagnosis between 2006 and 2007

Characteristics	Primary diagnosis (year)		
	1992–2009	2003–2009	2006–2007
Number of women	1798	825	242
Median age (years) (min.; max.)	24.0 (2.0; 74.0)	23.5 (2.0; 70.0)	23.9 (15.0; 70.0)
At least one recurrence	306 (17.0%)	141 (17.1%)	68 (28.1%)
One recurrence	220 (12.2%)	98 (11.9%)	44 (18.2%)
Two recurrences	58 (3.2%)	29 (3.5%)	11 (4.4%)
Three recurrences	19 (1.1%)	8 (1.0%)	7 (2.9%)
More than three recurrences	9 (0.5%)	5 (0.6%)	6 (2.5%)
Missing data	1 (0.1%)	1 (0.1%)	0 (0.0%)
Median time from primary treatment to first recurrence (weeks) (min.; max.)	14.6 (1.6; 701.0)	13.6 (1.6; 213.4)	12.7 (2.3; 126.7)

Highest percentage of recurrence was observed in the age group 10–15 years and percentage of recurrence decreased with increasing age (Fig. 1).

### Time to recurrence

Median time from primary treatment of genital warts with carbon dioxide laser to first recurrence was 14.6 (range 1.6–701.0) weeks. Median time from first to second and second to third recurrence was 16.6 (range 2.3–503.0) weeks and 21.3 (range 2.6–505.4) weeks, respectively. Of all women with at least one recurrence (*n* = 306), 44.6% experienced the recurrence within 12 weeks, 69.1% within six months and 82.9% within 1 year. In 8.1% of the study patients first recurrence was observed more than 3 years after primary treatment of genital warts.

### Additional data on topography, pregnancy status and cervical smear (data subset 1)

For the women who were treated for genital warts with carbon dioxide laser at our institution from 2003 to 2009 (*n* = 825; Table 1) additional information concerning topography of genitals warts, pregnancy status and cervical smear was available. In these 825 women, unifocal genital warts were observed in 195 (23.6%) and multifocal lesions in various combinations in 630 (76.4%) women (Table 2). The most frequently affected site (i.e. including uni- and multifocal warts) was the vulva (*n* = 748; 90.7%) followed by the perineum/perianal region (*n* = 489; 59.3%), the vagina (*n* = 390; 47.3%), the anus (*n* = 239; 29%) and the cervix (*n* = 110; 13.3%). Women with unifocal lesions had significantly fewer recurrences (11.3%) in comparison to women with multifocal lesions (18.9%) (*χ*^2^ = 6.08, *p* = 0.01). Additionally, increasing frequency of affected sites correlated with increasing percentage of recurrence. Of those women with genital warts in only one location 11.3% experienced a recurrence, whereas of those patients with four and five affected sites 21.8% and 24.0%, respectively, showed recurrences (Table 2). Anal genital warts were observed in 239 (28.9%) patients. In two (0.8%) of these 239 women additional lesions of the vagina or cervix were observed, whereas 99.2% (237 of 239) had additional lesions of the perineum, perianal region or vulva.

**Table 2:** Sites of distribution of unifocal and multifocal genital warts and recurrences (women with primary diagnosis of genital warts between 2003 and 2009)

Sites of distribution	Number of patients (%)	Recurrence (%)
All	825	141 (17.1%)
Unifocal	195 (23.6%)	22 (11.3%)
Vulva	150 (18.2%)	15 (10.0%)
Perineum/perianal	20 (2.4%)	2 (10.0%)
Vagina	18 (2.2%)	5 (27.8%)
Cervix	7 (0.9)	0 (0%)
Multifocal	630 (76.4%)	119 (18.9%)
Vulva–perineum/perianal	125 (15.2%)	22 (17.6%)
Vagina–vulva–perineum/perianal	100 (12.1%)	18 (18.0%)
Vagina–vulva	97 (11.8%)	15 (15.5%)
Vulva–perineum/perianal–anus	82 (9.9%)	14 (17.1%)
Vagina–vulva–perineum/perianal–anus	77 (9.3%)	18 (23.4%)
Cervix–vagina–vulva–perineum/perianal–anus	25 (3.0%)	6 (24.0%)
Cervix–vagina–vulva	23 (2.8%)	5 (21.7%)
Cervix–vagina–vulva–perineum/perianal	20 (2.4%)	4 (20.0%)
Perineum/perianal–anus	16 (1.9%)	2 (12.5%)
Cervix–vulva	11 (1.3%)	4 (36.4%)
Vagina–vulva–anus	10 (1.2%)	3 (30.0%)
Other combinations	44 (5.3%)	8 (18.2%)
Number of affected sites		
One site	195 (23.6%)	22 (11.3%)
Two sites	269 (32.6%)	45 (16.7%)
Three sites	226 (27.4%)	44 (19.5%)
Four sites	110 (13.3%)	24 (21.8%)
Five sites	25 (3.0%)	6 (24.0%)

Of the 825 patients with genital warts who underwent laser vaporisation between 2003 and 2009, 91 (11.0%) were pregnant. No significant differences in distribution of unifocal or multifocal genital warts were observed between pregnant and non-pregnant women (*χ*^2^ = 2.06, *p* = 0.15). Median week of gestation at the time of laser vaporisation was 21 (range 10–36) weeks. Recurrence rate in the 91 pregnant women was 18.7% (*n* = 17; non-pregnant patients 16.9%; *p* = 0.65). Of the 17 patients 15 (88.2%) experienced recurrence within the same pregnancy. Patients with a recurrence during pregnancy were treated earlier (median week of gestation: 16; range 10–33 weeks) than pregnant women without a recurrence (median week of gestation: 22; range 12–36 weeks; *U* = 414.0; *p* = 0.03).

Pap smear at time of primary treatment of genital warts was available for 712 (86.3%) of 825 women treated between 2003 and 2009. Abnormal cervical cytology was seen in 161 (22.6%) of these patients. The most frequent abnormal Pap smear was Pap IIID (64.6%) followed by Pap IIw (31.0%), Pap IV (2.5%) and Pap III (1.9%). Women with cervical genital warts significantly more frequently had abnormal Pap smears (43.7%) than did women with no involvement of the cervix (19.0%) (*χ*^2^ = 30.6, *p* < 0.001).

### Additional systematic follow-up data (data subset 2)

For women with genital warts treated between 2006 and 2007 (*n* = 242) systematic follow-up data were available. Median follow-up was 3.1 years (range 4 weeks to 5.9 years). Of these 242 women at least one recurrence was observed in 68 (28.1%) patients and more than three recurrences were seen in 2.5% (Table 1). Median time from primary treatment to first recurrence was 12.7 weeks (range 2.3 weeks to 2.4 years). First recurrence was observed within three months in 51.5% of these patients, while 86.8% of these patients showed the first recurrence within 12 months. Besides laser vaporisation, recurrence of genital warts was treated with imiquimod 5% cream (14.7%), excision (11.8%), podophyllotoxin (2.9%) and electrocoagulation (2.9%).

**Fig. 2:**
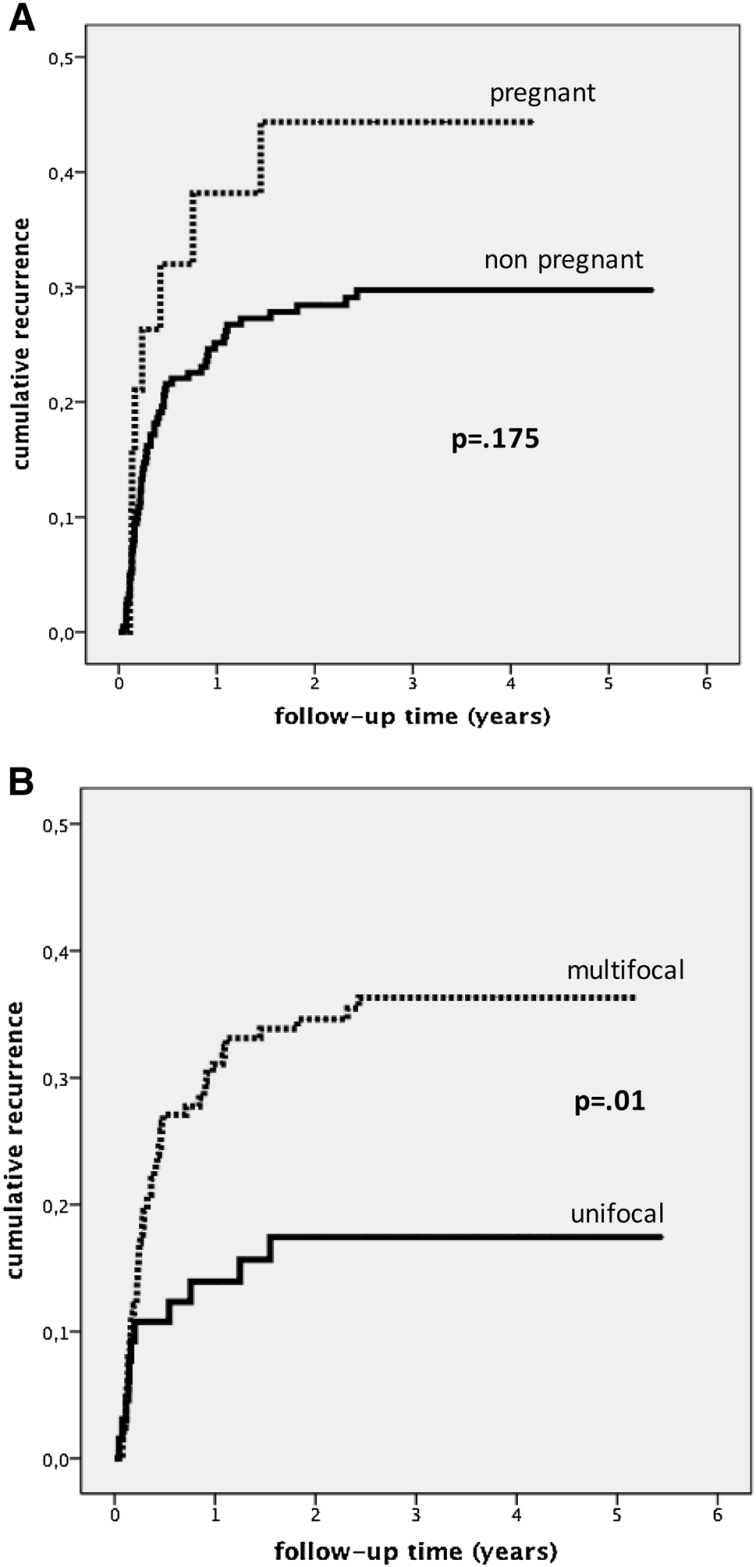
**a** Cumulative recurrence (Kaplan–Meier survival curves) for women treated between 2006 and 2007 (*n* = 242): Women pregnant vs non-pregnant at the date of treatment of genital warts. **b** Cumulative recurrence (Kaplan–Meier survival curves) for women treated between 2006 and 2007 (*n* = 242): multifocal vs unifocal genital warts

Pregnant women did not suffer significantly more often from recurrences than non-pregnant patients (*p* = 0.175, Fig. 2a).

Women with multifocal genital warts significantly more frequently had recurrences than did those with unifocal lesions (*p* = 0.01; Fig. 2b).

Multivariate analysis confirmed multifocal lesions as an independent risk factor for recurrence with a hazard ratio of 2.9 (Table 3). Specific locations of genital warts and age did not influence the risk of recurrence (Table 3). This analysis was performed on the subset of women treated in 2006 and 2007.

**Table 3:** Multivariate Cox regression model: risk factors for recurrence of genital warts

	Regression coefficient B	Wald	*df*	Sig	HR	95% Confidence Interval for HR	
						Lower	Upper
Number of affected sites (0 = unifocal, 1 = multifocal)	1.05	3.98	1	0.046	2.86	1.02	8.05
Site: Vagina	− 0.09	0.06	1	0.80	0.91	0.43	1.91
Site: Vulva	− 0.46	0.99	1	0.32	0.63	0.25	1.57
Site: Perineum/perianal region	− 0.11	0.09	1	0.77	0.89	0.43	1.87
Site: Anus	− 0.75	2.99	1	0.08	0.47	0.20	1.11
Age	− 0.03	2.23	1	0.14	0.97	0.93	1.01

*HR* hazard ratio, *Sig*. significance, *df* degrees of freedom

## Discussion

Nearly 30% of women with genital warts in the cohort with systematic follow-up data and 17% in the entire cohort presented with at least one recurrence after laser treatment. Median time to recurrence was 13–15 weeks, depending on the data subset. Nearly 87% of all recurrences occurred within the first year. Abnormal Pap smear was observed in nearly a quarter of patients. Multifocal lesions were found to be an independent predictor of recurrence.

### Increased incidence

Comparison of the analysed periods from 1992 to 2000 and from 2001 to 2009 showed an increase of 42% in new diagnosed cases of genital warts. Several other studies have also described an increased incidence and prevalence of genital warts within the past two decades [5, 7]. Possible reasons for this increase are changes in sexual behaviour [25], especially a greater number of sex partners, increased awareness, smoking [26], and oral contraceptive use [7]. On the other hand, implementation of the HPV vaccination has led to a decline of infection rates [22, 23, 27]. Both factors- an increase in the non- vaccinated population and a decline in the vaccinated population- underline the importance of the HPV vaccination.

### Recurrences

Analysis of all women showed at least one recurrence in 306 of 1798 patients (17%). Interestingly, 30% of the recurrences occurred after more than six months, suggesting that a short follow-up would dramatically underestimate the recurrence rate. It cannot be determined whether the specific recurrence was due to a new infection with a new HPV genotype, a re- infection with the former HPV genotype or a reactivation of the latent HPV genotype.

We collected systematic follow-up information from the gynecologist and/or general practitioner of each woman treated between 2006 and 2007 to estimate the true recurrence rate following laser treatment. These two years were chosen, because they were representative and allow a substantial follow-up period (median 3.1 years). At least one recurrence was observed in 28.1% of these women by contrast to 17.0% in the entire cohort. It is noteworthy that in about 13% of these women first recurrence was observed more than one year after primary treatment. This observation underlines the need to follow up women with genital warts for more than 1 year.

### Genital warts and cervical neoplasia

Genital warts have been suggested as a marker for exposure to HPV and subsequently for cervical neoplasia. Munk et al. reported a 1.9-fold increased risk for self-reported abnormal Pap smear in women with a history of genital warts [26]. Additionally, Friis et al. described a 2.0- and a 2.6-fold increased risk for cervical cancer and CIN III, respectively, for women following hospitalization for condylomata acuminate [28]. In the present study abnormal Pap smear was observed in 22.6% of our patients. Abnormal Pap smears were twice as frequent in women with cervical genital warts (43.7%) than in women without cervical involvement (19.0%) and were most frequent within the first two years of the first episode [26]. Based on the high percentage of abnormal Pap smears in women with genital warts and an increased risk for cervical neoplasia, Pap smear at the time of diagnosis of genital warts and regular Pap smear after treatment are recommended.

### Influencing factors

Little research has been conducted on the determinants influencing the recurrence of genital warts after primary treatment. In our study we observed a decrease in recurrences with increasing age. A possible explanation for this observation is that the number of sexual partners per year usually decreases with increasing age and therefore HPV viral load and HPV reinfections are less frequent.

### Multifocal genital warts

Women with multifocal genital warts had an almost three times higher risk of recurrence than women with unifocal lesions.

The most affected site was the vulva followed by the perineum/perianal region, the vagina and anus. About one-third of all investigated women had anal warts and almost all these patients additionally had warts in the perineum/perianal region or vulva. Given the fact that unknown anal warts are a frequent cause of relapse of genital warts, proctoscopic evaluation of all women with genital warts of the vulva or the perineum/perianal region is recommended to improve the management of anogenital warts. Proctoscopic examination would also allow the detection of dysplastic lesions in this area.

### Genital warts in pregnancy

In pregnancy treatment options for genital warts are limited. Topical treatment with podophyllotoxin or imiquimod cream is contraindicated. The most frequently recommended treatment strategies for pregnant women are surgical therapies including carbon dioxide laser vaporisation. In our cohort 11% of the women were pregnant and recurrence rates were comparable with those in non-pregnant patients. In accordance with Ferency et al. [29] we observed significantly fewer recurrences when laser vaporisation was performed in the third trimester than in the first or second trimester. Genital warts in pregnancy are a strong risk factor for juvenile-onset recurrent respiratory papillomatosis [30]. Due to the rarity of juvenile respiratory papillomatosis, there is no proof that treatment of genital warts in pregnancy reduces the risk of vertical HPV transmission. Nevertheless, treatment of genital warts in pregnancy and reduction of viral burden is recommended.

### Strengths and limitations

To the best of our knowledge this is the largest study of recurrence of genital warts in a not- vaccinated population. Main strengths are the large sample size, the period of time women were followed and the same treatment modality in all patients.

Our study encounters several limitations. Due to the retrospective design we cannot exclude a selection bias, although the large number of investigated women and the long follow-up could reduce a possible bias. Another weakness of this study is the lack of data on known risk factors for incident genital warts like sexual behaviour, smoking, and history of sexually transmitted diseases. The influence of these risk factors in the development of genital wart recurrence after primary treatment remains to be elucidated. In some cases we also lack information about former treatments.

## Conclusion

In summary, genital warts are an HPV-associated disease with increasing incidence. To the best of our knowledge this is the largest study investigating the incidence of genital wart recurrence after primary treatment with carbon dioxide laser in the pre-vaccination era. Nearly 30% of women with genital warts experience at least one recurrence (data subset 2), and median time from primary treatment to recurrence is 15 weeks in the entire cohort. Multifocal lesions are the strongest predictor for recurrence. Based on our data careful examination including Pap smear and proctoscopy as well as long-term follow-up after treatment are recommended to improve the management of genital warts. These data underline the importance of HPV vaccination especially in young women to reduce the incidence and consequently recurrence rates of HPV-associated genital warts.

## References

[1] Brown DR, Schroeder JM, Bryan JT (1999). Detection of multiple human papillomavirus types in condylomata acuminata lesions from otherwise healthy and immunosuppressed patients. J Clin Microbiol.

[2] Garland SM, Steben M, Sings HL (2009). Natural history of genital warts: analysis of the placebo arm of 2 randomized phase III trials of a quadrivalent human papillomavirus (types 6, 11, 16, and 18) vaccine. J Infect Dis.

[3] Wiley DJ, Douglas J, Beutner K (2002). External genital warts: diagnosis, treatment, and prevention. Clin Infect Dis.

[4] Hillemanns P, Breugelmans JG, Gieseking F (2008). Estimation of the incidence of genital warts and the cost of illness in Germany: a cross-sectional study. BMC Infect Dis.

[5] Kliewer EV, Demers AA, Elliott L (2009). Twenty-year trends in the incidence and prevalence of diagnosed anogenital warts in Canada. Sex Transm Dis.

[6] Saslow D, Andrews KS, Manassaram-Baptiste D (2016). Human papillomavirus vaccination guideline update: American Cancer Society guideline endorsement. CA Cancer J Clin.

[7] Kjaer SK, Tran TN, Sparen P (2007). The burden of genital warts: a study of nearly 70,000 women from the general female population in the 4 Nordic countries. J Infect Dis.

[8] Wiley D, Masongsong E (2006). Human papillomavirus: the burden of infection. Obstet Gynecol Surv.

[9] Dominiak-Felden G, Cohet C, Atrux-Tallau S (2013). Impact of human papillomavirus-related genital diseases on quality of life and psychosocial wellbeing: results of an observational, health-related quality of life study in the UK. BMC Public Health.

[10] Paradisi A, Capizzi R, Ricci F (2013). Quality of life in patients with anogenital warts. Eur J Dermatol.

[11] Jeynes C, Chung MC, Challenor R (2009). “Shame on you–”the psychosocial impact of genital warts. Int J STD AIDS.

[12] Park IU, Introcaso C, Dunne EF (2015). Human papillomavirus and genital warts: a review of the evidence for the 2015 centers for Disease Control and Prevention Sexually Transmitted Diseases Treatment Guidelines. Clin Infect Dis.

[13] Scheinfeld N, Lehman DS (2006). An evidence-based review of medical and surgical treatments of genital warts. Dermatol Online J.

[14] Lacey CJN, Woodhall SC, Wikstrom A, Ross J (2013). 2012 European guideline for the management of anogenital warts. J Eur Acad Dermatol Venereol.

[15] Savoca S, Nardo LG, Rosano TF (2001). CO(2) laser vaporization as primary therapy for human papillomavirus lesions. A prospective observational study. Acta Obstet Gynecol Scand.

[16] Duus BR, Philipsen T, Christensen JD (1985). Refractory condylomata acuminata: a controlled clinical trial of carbon dioxide laser versus conventional surgical treatment. Genitourin Med.

[17] Chen K, Chang BZ, Ju M (2007). Comparative study of photodynamic therapy vs CO2 laser vaporization in treatment of condylomata acuminata: a randomized clinical trial. Br J Dermatol.

[18] Aynaud O, Buffet M, Roman P (2008). Study of persistence and recurrence rates in 106 patients with condyloma and intraepithelial neoplasia after CO2 laser treatment. Eur J Dermatol.

[19] Ferenczy A, Behelak Y, Haber G (1995). Treating vaginal and external anogenital condylomas with electrosurgery vs CO2 laser ablation. J Gynecol Surg.

[20] Krebs HB, Wheelock JB (1985). The CO2 laser for recurrent and therapy-resistant condylomata acuminata. J Reprod Med.

[21] Thomas R, Steben M, Greenwald Z (2017). Recurrence of human papillomavirus external genital wart infection among high-risk adults in Montréal, Canada. Sex Transm Dis.

[22] Ali H, Donovan B, Wand H (2013). Genital warts in young Australians five years into national human papillomavirus vaccination programme: national surveillance data. BMJ.

[23] Drolet M, Bénard É, Boily M-C (2015). Population-level impact and herd effects following human papillomavirus vaccination programmes: a systematic review and meta-analysis. Lancet Infect Dis.

[24] Soost HJ (1993). The Munich nomenclature. Recent Results Cancer Res.

[25] Habel LA, Van Den Eeden SK, Sherman KJ (1998). Risk factors for incident and recurrent condylomata acuminata among women. A population-based study. Sex Transm Dis.

[26] Munk C, Svare EI, Poll P (1997). History of genital warts in 10,838 women 20 to 29 years of age from the general population. Risk factors and association with Papanicolaou smear history. Sex Transm Dis.

[27] Drolet M, Laprise J-F, Brotherton JML (2017). The impact of human papillomavirus catch-up vaccination in Australia: implications for introduction of multiple age cohort vaccination and postvaccination data interpretation. J Infect Dis.

[28] Friis S, Kjaer SK, Frisch M (1997). Cervical intraepithelial neoplasia, anogenital cancer, and other cancer types in women after hospitalization for condylomata acuminata. J Infect Dis.

[29] Ferenczy A (1984). Treating genital condyloma during pregnancy with the carbon dioxide laser. Am J Obstet Gynecol.

[30] Silverberg MJ, Thorsen P, Lindeberg H (2003). Condyloma in pregnancy is strongly predictive of juvenile-onset recurrent respiratory papillomatosis. Obstet Gynecol.

